# Resistance Mutations in *gyrA* and *parC* are Common in *Escherichia* Communities of both Fluoroquinolone-Polluted and Uncontaminated Aquatic Environments

**DOI:** 10.3389/fmicb.2015.01355

**Published:** 2015-12-09

**Authors:** Anna Johnning, Erik Kristiansson, Jerker Fick, Birgitta Weijdegård, D. G. Joakim Larsson

**Affiliations:** ^1^Department of Infectious Diseases, Institute of Biomedicine, University of GothenburgGothenburg, Sweden; ^2^Department of Mathematical Sciences, Chalmers University of TechnologyGothenburg, Sweden; ^3^Department of Chemistry, Umeå UniversityUmeå, Sweden

**Keywords:** antimicrobial agents, mechanisms of resistance, antibiotics, microbial communities, next generation sequencing

## Abstract

Alterations in the target proteins of fluoroquinolones, especially in GyrA and ParC, are known to cause resistance. Here, we investigated environmental *Escherichia* communities to explore the possible link between the abundance of mutations, and the exposure to fluoroquinolones. Sediment samples were collected from a relatively pristine lake, up and downstream from a sewage treatment plant, and from several industrially polluted sites. The quinolone resistance-determining regions of *gyrA* and *parC* were analyzed using amplicon sequencing of metagenomic DNA. Five non-synonymous substitutions were present in all samples, and all of these mutations have been previously linked to fluoroquinolone resistance in *Escherichia coli*. In GyrA, substitutions S83L and D87N were on average detected at frequencies of 86 and 32%, respectively, and 31% of all amplicons encoded both substitutions. In ParC, substitutions S80I, E84G, and E84V were detected in 42, 0.9, and 6.0% of the amplicons, respectively, and 6.5% encoded double substitutions. There was no significant correlation between the level of fluoroquinolone pollution and the relative abundance of resistance mutations, with the exception of the most polluted site, which showed the highest abundance of said substitutions in both genes. Our results demonstrate that resistance mutations can be common in environmental *Escherichia*, even in the absence of a fluoroquinolone selective pressure.

## Introduction

Fluoroquinolones are a class of synthetic broad-spectrum antibiotics that target the type II topoisomerases (DNA gyrase and topoisomerase IV) involved in the maintenance of DNA topology. DNA gyrase and topoisomerase IV are both tetrameric enzymes composed of two X and two Y subunits (X_2_Y_2_). DNA gyrase is encoded by the genes *gyrA* and *gyrB*, and topoisomerase IV is encoded by *parC* and *parE*. The enzymes are homologues, and there is a considerable sequence similarity between *gyrA* and *parC*, and between *gyrB* and *parE*. One of the main mechanisms of fluoroquinolone resistance is amino acid substitutions in the DNA gyrase and topoisomerase IV proteins, in particular in GyrA and ParC (Ruiz, 2003; Hopkins et al., 2005). Several resistance mutations have been characterized in *Escherichia coli*, and the majority of these are located in the quinolone resistance-determining region (QRDR) defined as codons 67–106 in *gyrA* and 56–108 in *parC* (*E. coli* numbering). Certain single mutations in *gyrA* are sufficient to generate high-level resistance to nalidixic acid, a non-fluorinated first generation quinolone. Additional mutations in *gyrA* or other type II topoisomerase genes are, however, necessary for high-level resistance to later generations of fluoroquinolones, such as ciprofloxacin (Hopkins et al., 2005). Indeed, a study of 58 fluoroquinolone-resistant clinical *E. coli* isolates from two hospitals in Houston, TX, USA, found that all isolates had amino acid substitutions in GyrA and approximately 85% had additional substitutions in ParC (Morgan-Linnell et al., 2009), confirming chromosomal mutations as the main mechanism of clinically relevant fluoroquinolone resistance. The frequency of resistant invasive *E. coli* infections in Europe in 2012 ranged from 9.7% in Iceland to 42.0% in Cyprus (ECDC, 2013). Surveillance outside of Europe is less systematic, but a hospital in India reported that as many as 73% of uropathogenic *E. coli* infections were resistant to ciprofloxacin (Mandal et al., 2012).

*Escherichia coli* primarily propagate in the intestines of warm blooded animals, but also, to a limited extent, in the environment (Ishii and Sadowsky, 2008). Because fluoroquinolones are strictly synthetic antibiotics, it would be reasonable to assume that resistance mutations are not as common in environmental *E. coli* isolates as they are among clinical isolates. Accordingly, screening of environmental bacteria from an isolated cave for antibiotic resistance showed very few isolates that were resistant to a high concentration of ciprofloxacin (MIC >20 mg/l), whereas resistance to other classes of antibiotics were more common (Bhullar et al., 2012). Similarly, a recent study from our group found that only <1–6% of bacteria isolated from lakes with no documented history of fluoroquinolone pollution, in Sweden and India, were ciprofloxacin-resistant (MIC >2 mg/l) (Flach et al., 2015). A characterization of the QRDR of *gyrA* from 20 environmental *E. coli* isolates showed that there was considerable variability in the amino acid sequence (Waters and Davies, 1997), and a study of 38 highly ciprofloxacin-resistant (MIC 6–128 mg/l) soil-dwelling bacterial isolates of different species showed that nine isolates (24%) contained amino acid substitutions in this region (D’Costa et al., 2006). This suggests that mutations in the QRDR can be present in environmental bacteria even in the absence of fluoroquinolones. The methods used in previous studies have, however, been low throughput, making the estimation of the abundance of mutations unreliable. Previous studies have also been dependent on the culturability of the bacteria, and often focused on phenotypically fluoroquinolone-resistant bacteria (Adachi et al., 2013; Zurfluh et al., 2014). The characteristics and relative abundance of resistance mutations in the QRDRs in environmental bacterial communities, have, thus remained unknown.

Recently, concerns have been raised about the risks of resistance development due to antibiotic contamination of the external environment (Gaze et al., 2013; Pruden et al., 2013; Wellington et al., 2013). Antibiotics, including fluoroquinolones, can enter the environment through, for example, human sewage and use in animal farming (Kümmerer, 2009). The highest concentrations of fluoroquinolones detected in the environment are, however, the result of direct discharge from manufacturing (Larsson et al., 2007; Fick et al., 2009; Kristiansson et al., 2011). It is therefore important to assess the risks of the development and spread of resistant bacteria in contaminated environments. Furthermore, because the discharge of antibiotics may be intermittent and difficult to detect, acquired resistance characteristics of environmental bacteria may, given sufficient evaluation, serve as sentinels and biomarkers for antibiotic exposure. The presence and abundance of *qnr*-genes, a group of mobile fluoroquinolone resistance genes, in microbial communities have been shown previously to correlate with fluoroquinolone contamination of stream sediments (Kristiansson et al., 2011). However, horizontally transferred genes are not ubiquitous and may, therefore, not be optimal as biomarkers. In contrast, chromosomal resistance mutations can appear *de novo* and be enriched in a population under sufficient antibiotic selective pressure. Hence, we hypothesize that resistance mutations in *gyrA* and *parC* could serve as biomarkers for fluoroquinolone exposure in different environments.

The aim of the present study was to assess the abundance of chromosomal fluoroquinolone resistance mutations in environmental *Escherichia* communities and to investigate the potential link to fluoroquinolone pollution. Using massively parallel amplicon sequencing of metagenomic DNA, we determined the full genetic resistance characteristics of the QRDR of *gyrA* and *parC* in *Escherichia* communities without any prior culturing of bacteria. The studied environments included a range of selective pressures for fluoroquinolone resistance from a highly polluted Indian stream, samples taken up and downstream from a Swedish sewage treatment plant, and from a remote small Swedish lake under minimal human impact. This allowed us to investigate a possible correlation between fluoroquinolone pollution and the abundance of resistance mutations. Our analysis showed a high abundance of fluoroquinolone resistance mutations in both *gyrA* and *parC* in all investigated environments, but, interestingly, we did not find any significant correlation with the levels of fluoroquinolones detected.

## Materials and Methods

### Sample Collection

Samples were taken from an Indian stream and a Swedish stream both upstream (two in India, one in Sweden) and downstream (three in India, one in Sweden) of wastewater treatment plants (WWTPs) as described previously (Kristiansson et al., 2011). The Indian WWTP is situated in Patancheru near Hyderabad, India, and at the time of sampling, it received industrial eﬄuent from approximately 90 pharmaceutical industries. The Swedish WWTP is located in Skövde and receives municipal wastewater, but with no input from pharmaceutical industries. These seven samples have previously been analyzed for the presence of mobile antibiotic resistance genes using metagenomic sequencing (Kristiansson et al., 2011), and *qnr* genes using qPCR (Rutgersson et al., 2014). For this study, three additional sediment samples were collected from an upland Swedish small lake, Valbergs öga, situated far from habitation, roads and farmland and with no apparent inflow of water (2012-07-25, GPS coordinates 57°51′34.2″N 12°04′58.2″E). Although few places on earth could be referred to as completely pristine, very few people, if any, are likely to walk near this small lake in a given year. Thus, it represents an environment at the very low end in terms of human impact. To determine the fluoroquinolone selective pressure at the samplings sites, the concentrations of ciprofloxacin, difloxacin, enoxacin, enrofloxacin, lomefloxacin, ofloxacin, pefloxacin, and norfloxacin were measured in all samples using liquid chromatography coupled to an ion trap mass spectrometer and electro spray interface (LC-ESI-IT-MSMS) described previously by Kristiansson et al. (2011), where the original chemical analysis data of all of the stream sediment samples can be found. The Swedish lake samples were analyzed at a different time point using the same method. Results were expressed per gram organic matter, rather than per total weight as some samples contained a large proportion of inorganic material (gravel). The detection limit was 0.02 μg/g organic matter for each measured substance.

### DNA Extraction

The total DNA was extracted from the sediment samples and amplified using uniform whole genome amplification. A PowerSoil^®^ DNA Isolation Kit (MO BIO Laboratories, Inc., Carlsbad, CA, USA) was used for the DNA extraction with the following modification to the manufacturer’s protocol: to complete homogenization and cell lysis, the power bead tubes with sediment and solution C1 were incubated at 70°C for 10 min with a brief vortex after 5 and 10 min. The DNA concentration was measured using a NanoDrop (Thermo Fisher Scientific Inc., Waltham, MA, USA), and the samples were stored at -80°C until the whole genome amplification of purified genomic DNA was performed. Uniform whole genome amplification was done using the REPLI-g mini kit (QIAGEN, Hilden, Germany). The DNA (10 ng) was denatured by adding a denaturation buffer and incubating for 3 min at room temperature. The denaturation was interrupted by a neutralization buffer and a master mix containing a reaction buffer, and DNA polymerase was added. All buffers and enzyme mixtures were made according to the protocol. The amplification reaction was performed by incubating the samples at 30°C for 16 h in a thermal cycler, after which the reaction was stopped by heating the sample for 3 min at 65°C. The DNA concentration was measured using a NanoDrop, and the samples were stored at -20°C before the PCR amplification of the target genes.

### PCR Amplification of *gyrA* and *parC*

The software Primer3 (Rozen and Skaletsky, 2000) was used through the web interface Primer3Plus (Untergasser et al., 2007) to design PCR primers targeting the regions of *gyrA* and *parC* that include all resistance mutations reported by Ruiz (2003). To avoid placing primers in variable regions, all annotated *gyrA* and *parC* nucleotide sequences belonging to *Escherichia* were downloaded from the Pathosystems Resource Integration Center (PATRIC) (Gillespie et al., 2011) and each gene was aligned using MUSLCE (Edgar, 2004). Because *Shigella* is indistinguishable from *Escherichia* in the targeted gene regions, this genus was also added to the multiple sequence alignment. All variable positions were given as excluded regions in Primer3, and the portions including all resistance mutations were given as the target regions. The product size range was set as 250–350 base pairs (bps) so that the amplicons would, to a large extent, be completely covered by a single 454 sequence read. The top 10 suggested primers were tested experimentally to determine the best primer pair, producing the largest amount of amplicons of the expected length, for each gene. The selected primers, targeted regions, and experimental design are shown in **Table 1.**

**Table 1:** Selected primers with target regions and experimental setup.

Gene	Target region	Product size	Forward primer	Reverse primer	Annealing temperature
*gyrA*	129–439	311 bp	ggtacaccgtcgcgtacttt	caacgaaatcgaccgtctct	57°C
*parC*	20–306	287 bp	gccttgcgctacatgaattt	accatcaaccagcggataac	57°C


All of the samples were amplified using the selected primer pair for each gene and the set-up described in **Table 1.** The following was combined in sterile 0.2 ml tubes: 1xGotaq^®^Reaction buffer, 0.2 mM dNTP Mix, 0.4 μM forward primer, 0.4 reverse primer, 1.25 μ GoTaq^®^ DNA polymerase (Promega, Madison, WI, USA), 50 ng template, and water up to 25 μl. The PCR amplification used an initial denaturation at 95°C for 2 min, denaturation at 95°C for 10 s, annealing at 57°C for 30 s, extension at 72°C for 1 min, and a final extension for 7 min. Each reaction was run for 40 cycles to maximize the amount of amplicon DNA. The PCR products were separated using 1.5% agarose gel electrophoresis and visualized with ethidium bromide. The DNA fragment of interest was excised from the agarose gel and placed in a microcentrifuge tube, and the GeneJET^TM^ gel extraction kit (Fermentas International Inc., Vilnius, Lithuania) was used according to the enclosed protocol to obtain the DNA amplicons. The purified amplicons were pooled to enable sequencing in a single run, and therefore, each sample contained amplicons of both *gyrA* and *parC* derived from the same sample site. The amplicons were stored at -20°C before sequencing.

### DNA Sequencing and Bioinformatics Analyses

The samples were sent to GATC Biotech (Konstanz, Germany) for multiplexed massively parallel pyrosequencing (Margulies et al., 2005) using titanium chemistry on the GS FLX+ system. The sequencing of the Swedish lake samples was performed on a separate time point. The resulting sequence reads were aligned to their respective reference sequence belonging to *E. coli* K-12 MG1655 (RefSeq locus tags, *gyrA*: b2231; *parC*: b3019) using GS Amplicon Variant Analyzer from 454 (v2.5p1). The aligned reads were exported from the software as one FASTA alignment file per gene and sample site. To limit the risk of interpreting sequence variability between species as resistance mutations, reads too dissimilar to the *Escherichia* and *Shigella* sequences were discarded after matching all reads against all sequences annotated with the gene symbol *gyrA* and *parC*, respectively, in the Comprehensive Microbial Resource (CMR) (Peterson et al., 2001) using BLASTn (Altschul et al., 1990). Only reads with a hit against a *gyrA* or *parC* sequence from either *Escherichia* or *Shigella*, and with an *E*-value lower than 1 × 10^-100^ were retained in the subsequent analysis. To compensate for the issue of homopolymers, which are common in 454 sequencing, any positions in the multiple sequence alignments with insertions in the reference sequence were removed, and any remaining deletions in the reads were substituted with the reference sequence base of that position. The resulting gapless reads were translated using the EMBOSS tool Transeq (reading frame 1 for *gyrA* and 2 for *parC*) (Rice et al., 2000). The relative abundance of non-synonymous substitutions was recorded. The relative abundance of a certain mutation was defined as the percentage of reads encoding that particular amino acid substitution. The average relative abundance of mutations was defined as the percentage of reads encoding any amino acid substitution averaged over all samples. The computations were done for both individual substitutions, and pairs of substitutions occurring within the same read. The correlation between fluoroquinolone pollution – measured as the total amount of fluoroquinolones detected (log-transformed) – and the abundance of resistance mutations was measured using Pearson’s correlation coefficient.

#### Nucleotide Sequence Accession Number

The raw sequence data have been submitted to the Short Read Archive (SRA) under BioProject accession number PRJNA239415.

## Results

The measurements of the fluoroquinolone concentrations present in the sediments confirmed that the sampled environments represented a range of different fluoroquinolone selective pressures (see **Supplementary Table S1**). Ciprofloxacin, enrofloxacin, and pefloxacin were detected at all Indian sample sites, ofloxacin was detected only upstream from the Indian WWTP, and lomefloxacin and difloxacin were detected only at the Indian downstream sites. None of the analyzed fluoroquinolones were detected in any of the Swedish samples. The highest level of fluoroquinolones was detected in the Indian downstream samples, while the upstream samples contained moderately high levels, as reported previously (Kristiansson et al., 2011).

A total of 35,417 reads aligned to the *gyrA* reference sequence (1,453–6,339 reads per sample), and 51,933 reads aligned to the *parC* reference sequence (537–15,469 reads per sample) (**Table 2**). The filtering of reads dissimilar to known *Escherichia* and *Shigella* sequences retained on average 60% of the *gyrA* reads (45–91% per sample) and 78% of the *parC* reads (72–95% per sample), suggesting that the *parC* primer pair is more specific for the targeted genera in the sampled environments compared to the *gyrA* primers. For *gyrA*, a total of 21,295 reads were used in the subsequent analysis (790–3,104 reads per sample), and 40,521 reads were used for *parC* (509–12,006 reads per sample).

**Table 2:** Number of reads that aligned and that was retained after quality filtering.

Country	Site	Sample	Aligned reads		Filtered reads	
				
			GyrA	ParC	GyrA	ParC
India	Downstream	1	3,051	712	2,281	658
		2	1,531	3,759	790	3,425
		3	2,788	537	1,753	509
	Upstream	1	3,409	766	3,104	658
		2	3,057	1,919	1,838	1,664
Sweden	Downstream		1,453	2,079	888	1,831
	Upstream		4,425	2,926	2,773	2,112
	Lake	1	4,672	15,469	2,438	12,006
		2	4,692	12,446	2,609	9,039
		3	6,339	11,320	2,821	8,619
		**Sum**	35,417	51,933	21,295	40,521


**FIGURE 1:**
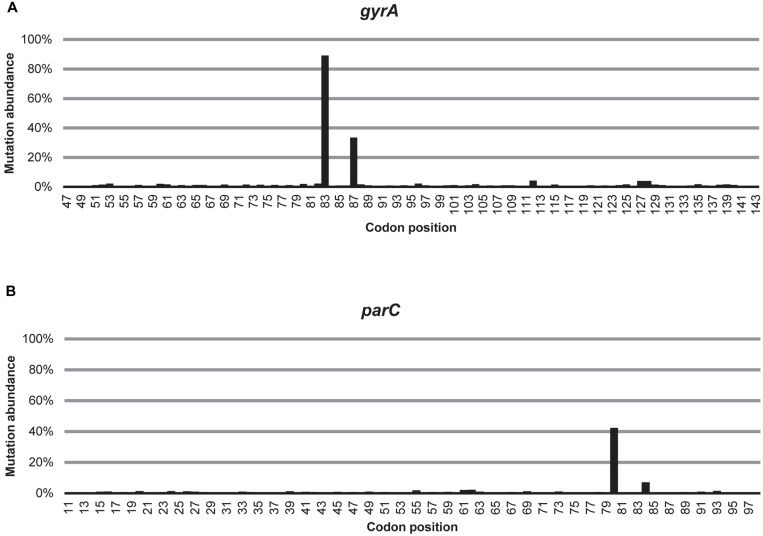
**Detected average variability over the amplicon.** The average relative abundance of non-synonymous substitutions in each codon of **(A)** the *gyrA* and **(B)** the *parC* amplicons, averaged over all samples.

Out of all the non-synonymous substitutions detected, the variability in codon 83 and 87 in *gyrA* and in codon 80 in *parC* was considerably higher than at any other codon (**Figure 1**). Three non-synonymous mutations in *gyrA* were detected in all samples and they encode the following amino acid substitutions: S83L, D87N, and D82G (see **Supplementary Table S2a**). The most common substitution, S83L, was detected in a majority of reads in all samples (61–97%), while D87N was less abundant (1–62%). Almost all of the D87N substitutions (97%) occurred in reads also encoding the S83L substitution. Although the D82G substitution was detected in all samples, it was rare (≤1%), as were other resistance mutations detected in only some of the samples (0–2%). Three non-synonymous mutations in *parC* were detected in all samples and they encode the following amino acid substitutions: S80I, E84G, and E84V (see **Supplementary Table S2b**). S80I was the most common substitution encoded by the *parC* amplicons, and was detected in 0.4–63% of the reads in each sample. The mutations in codon 84 were far less common, but 98% of all reads carrying substitutions in codon 84 also carried mutations encoding the S80I substitution. Other resistance mutations in *parC*, which were detected in only some of the samples, were rare (0–7%).

**FIGURE 2:**
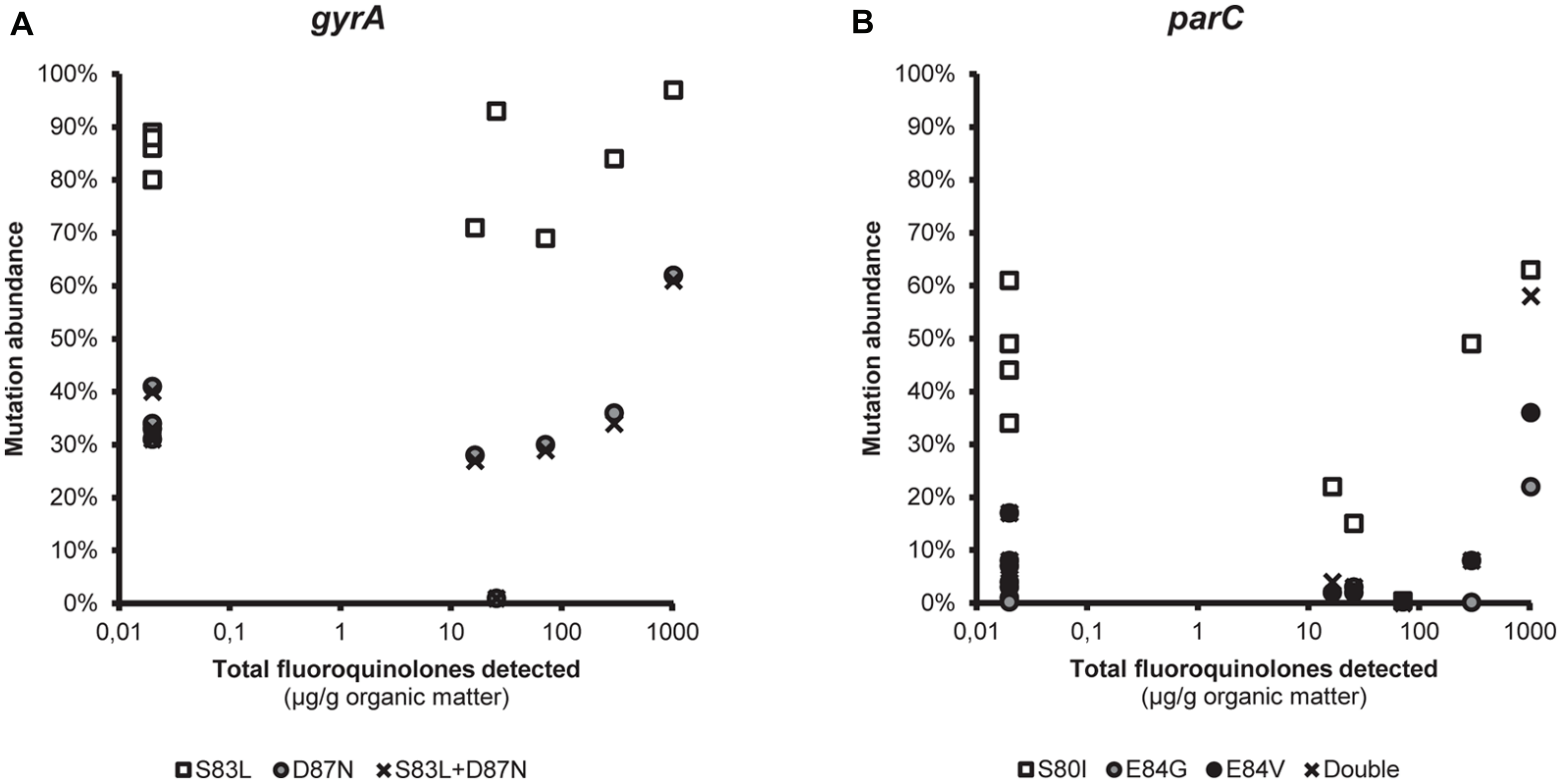
**The link between fluoroquinolone pollution and mutations in the quinolone resistance determining region.** Correlation between the measured total fluoroquinolone pollution (μg/g organic matter) and the relative abundance of the most commonly detected non-synonymous substitutions in **(A)** *gyrA* and **(B)** *parC*. Samples with no detected fluoroquinolones are indicated in the figure as 0.2 μg/g (i.e., the detection limit).

The differences in terms of non-synonymous mutations between the environments were small, and there was no obvious link between the abundance of these mutations and the fluoroquinolone selective pressure for either *gyrA* or *parC* (**Figure 2**, also see **Supplementary Figures S1** and **S2**). Pearson’s correlation coefficient between the log-transformed detected amounts of total fluoroquinolones (samples with no detected fluoroquinolones were set to the detection limit 0.02 μg/g organic matter) and the abundance of the most commonly detected substitutions were as follows: S83L, -0.058 (*p* = 0.87); D87N, 0.098 (*p* = 0.79); and S83L+D87N in GyrA, 0.088 (*p* = 0.81); S80I, -0.25 (*p* = 0.49); E84G, 0.54 (*p* = 0.10); E84V, 0.28 (*p* = 0.42); and S08I+E84G/V in ParC, 0.34 (*p* = 0.26). However, the sample taken downstream from and closest to the discharge site of the Indian WWTP showed the highest abundance of all said substitutions (**Figure 2**, also see **Supplementary Figures S1a** and **S2a**).

## Discussion

In this study, we measured the relative abundance of chromosomal fluoroquinolone resistance mutations in *Escherichia* communities residing in both uncontaminated and severely fluoroquinolone polluted aquatic environments. We detected a high abundance of resistance mutations in the chromosomal target genes of fluoroquinolones, *gyrA* and *parC*, in all sampled environments, including environments with no fluoroquinolone pollution or known history thereof. Interestingly, we found no association between the measured concentrations of fluoroquinolones at the samples sites and the abundance of resistance mutations, except for the most polluted site, which showed the highest abundance of the most commonly detected mutations. This suggests that chromosomal mutations conferring fluoroquinolone resistance only could be considered a potential biomarker for detecting very extensive fluoroquinolone pollution. Additionally, the high abundance of mutations previously linked to fluoroquinolone resistance and the low abundance of other mutations indicate that there could be selective advantages to carry the resistance mutations in the sampled environments, even in the absence of fluoroquinolone selective pressure. Note that the levels of fluoroquinolones detected in the sediment cannot be directly compared to those of water solutions, as some fluoroquinolones are known to sorb to soil particles (Girardi et al., 2011). The exact amount of bioavailable antibiotics is, therefore, unknown, but likely lower than the detected levels.

To our best knowledge, the lowest reported minimal selective concentration for any antibiotic and resistance mutation is 0.1 μg/l for ciprofloxacin and the S83L substitution in GyrA (Gullberg et al., 2011). This concentration is just above the ones found in treated Swedish sewage eﬄuents (Lindberg et al., 2005; Fick et al., 2011). However, the concentration was determined through competition experiments in a lab setting, and therefore, comparisons to environmental conditions should be made with some caution. Although no fluoroquinolones were detected downstream from the Swedish sewage treatment plant in this study, bacteria leaving the plant could theoretically have encountered selective concentrations inside the treatment plant. In the investigated Swedish lake, however, there are no plausible sources for fluoroquinolone exposure, indicating that the mutations found here are not present because of a fluoroquinolone selection pressure. Furthermore, since there is little difference in mutation abundance between the severely polluted sites and the remote Swedish lake, the data implies that the mutation abundance is not connected to fluoroquinolone selection pressure, but is largely determined by other, unknown factors.

In *gyrA*, mutations encoding the amino acid substitutions S83L and D87N were detected in all samples, with almost all of the D87N substitutions occurring in reads also encoding the S83L substitution. Both substitutions have been associated with fluoroquinolone resistance for *E. coli* and confer higher levels of resistance than any other substitutions in the QRDR (Yoshida et al., 1991). In fluoroquinolone-resistant clinical *E. coli* isolates, S83 is the most frequently altered amino acid, and, moreover, S83L is the most common substitution (Hopkins et al., 2005). A single substitution at position 83 confers higher resistance than at position 87, but additional mutations in *gyrA* or other genes are necessary for the development of high-level fluoroquinolone resistance. The S83L substitution alone, however, confers the same level of resistance to ciprofloxacin for *E. coli* as the combination of S83L and D87N (increased MIC of ciprofloxacin from 0.016 to 0.38 mg/l), while the D87N substitution alone confers slightly lower resistance (ciprofloxacin MIC = 0.25 mg/l) (Marcusson et al., 2009; Machuca et al., 2014). In *parC*, mutations causing the substitutions S80I, E84G, and E84V were detected in all samples, and these three substitutions have been previously linked to fluoroquinolone resistance in *E. coli*. In fluoroquinolone-resistant clinical isolates, the most commonly altered amino acid in ParC is S80 followed by E84, and, specifically, S80I is the most common substitution (Hopkins et al., 2005). Substitutions in ParC are often, if not always, found together with substitutions in GyrA in fluoroquinolone-resistant isolates (Hopkins et al., 2005; Morgan-Linnell et al., 2009), and substitutions of S80 or E84 alone do not affect the susceptibility to fluoroquinolones unless GyrA is also altered (Bagel et al., 1999). Because of the method used in this study, it is not possible to determine to what extent the detected mutations in *gyrA* and *parC* occur in the same bacterium, but it is worth mentioning that a combination of the substitutions S83L and D87N in GyrA with S80I in ParC confers a very high resistance to ciprofloxacin (ciprofloxacin MIC = 32 mg/l) (Marcusson et al., 2009). To set the MICs into perspective, an Enterobacteriaceae isolate is classified as ciprofloxacin-resistant if its MIC is higher than 1 mg/l according to the EUCAST clinical breakpoints (EUCAST, 2014). Isolates with a MIC lower than 0.5 mg/l are classified as sensitive. It is important to note that fluoroquinolone resistance can be caused by a number of different genetic mechanisms and combinations thereof. To extrapolate a level of resistance solely from mutations in the target genes is associated with a high level of uncertainty, therefore, the MICs given above are only included as a rough reference.

The only sampling site that stood out in terms of high abundance of resistance mutations was the site closest to downstream of the discharge site of the Indian WWTP. Here, we observed the highest abundance of the S83L and D87N substitutions in GyrA, the S80I, E84G, and E84V substitutions in ParC, and, consequently, also the highest abundance of the paired substitutions in both proteins. This is indeed the sample site with the highest total concentration of fluoroquinolones, which would be consistent with the hypothesis that there is a selective advantage for resistance mutations in fluoroquinolone-polluted environments, but only at locations with very high concentrations of fluoroquinolones. However, some reads from the most polluted site did not contain any mutations that resulted in amino acid substitutions in either *gyrA* (1.2%) or *parC* (30%), which suggests that some bacteria thriving there may carry additional resistance factors protecting them. Indeed, we have shown previously that mobile fluoroquinolone-resistance genes (*qnr*) are highly abundant in the stream sediment (Rutgersson et al., 2014). The *qnr* genes provide low to moderate resistance to fluoroquinolones [ciprofloxacin MIC up to 2 mg/l in *E. coli* transconjugants (Robicsek et al., 2006; Flach et al., 2013)]. Given the data presented here, it is possible that the presence of *qnr* genes in aquatic bacterial comminutes constitutes a better marker of fluoroquinolone exposure than chromosomal mutations. Or, there could be other mutations, outside the QRDR sequenced in this study, better suited as markers of exposure. Culturing-based methods will be required to fully elucidate the genetic mechanisms for how bacteria have adapted to survive the high concentrations of fluoroquinolones here.

A contributing factor to the high abundance of fluoro quinolone resistance mutations in the environment could be that there is little to no cost for the bacterium to carry these mutations. It has previously been shown that the substitutions S83L and D87N in GyrA and S80I in ParC occurring alone, or all three in the same organism, do not significantly alter the fitness of *E. coli in vitro*, nor do the pair of S83L in GyrA and S80I in ParC (Marcusson et al., 2009; Machuca et al., 2014). The pair S83L and D87N in GyrA has been shown to confer a fitness cost (measured relative fitness 0.97) when introduced in *E. coli* K-12 MG1655 (Marcusson et al., 2009), while the pair substitution was associated with a fitness gain in *E. coli* ATCC 25922 (relative fitness 1.14) (Machuca et al., 2014). In contrast, the pair of D87N in GyrA together with S80I in ParC introduce a slight fitness gain (measured relative fitness 1.02). Competition experiments with wild type *gyrA* in *E. coli* and mutants encoding either the S83L or the D87N substitution have shown that a ciprofloxacin concentration of 0.1 and 2.5 μg/l, respectively, is sufficient for the mutants to have a selective advantage (Gullberg et al., 2011). Furthermore, competitive i*n vivo* studies of other species – i.e., *Neisseria gonorrhoeae* in mice (Kunz et al., 2012) and *Campylobacter jejuni* in chickens (Luo et al., 2005) – have even found a fitness benefit associated with *gyrA* mutations corresponding to codon 83 in *E. coli*. In summary, a lack of fitness cost for these substitutions in the sampled environments could explain why they appear to be very common even in environments without any detectable fluoroquinolone pollutants or any known history thereof. As we do not yet understand the possible advantages these genotypes may have in the absence of fluoroquinolones, and because of the limited number and types of environments investigated in this study, we cannot generalize to the point of claiming that resistance mutations in *gyrA* and *parC* are common in all environmental *Escherichia* communities.

The abundance of resistance mutations in the Swedish samples stands in contrast to the frequency of fluoroquinolone-resistant infections in Sweden. On average, the S83L substitution in GyrA was detected in 87% of the Swedish reads, the combination of S83L and D87N in 33%, and the S80I substitution in ParC in 43%. In Sweden, only 11.6% of the clinical invasive *E. coli* isolates tested with ciprofloxacin were resistant in 2013 (ECDC, 2014). This is far below the abundance of resistance mutations detected in both the Swedish lake samples and the samples taken near a sewage treatment plant. The large difference between the abundance of fluoroquinolone resistance mutations in the environment and the frequency of resistant *E. coli* infections in Sweden is likely due to large differences between the pathogenic *E. coli* strains and the strains residing in the sampled environments. Indeed, the genome of *E. coli* is known to be highly variable (Lukjancenko et al., 2010). Furthermore, competition experiments testing the relative fitness of the resistance mutations is typically performed under optimal growing conditions, unlike environmental conditions. It is possible that there is a selective advantage not linked to fluoroquinolones for these mutations in the sampled environments. Indeed, our finding of how abundant the resistance mutations are even in unpolluted environments suggest that the sequence defined as wild-type for GyrA and ParC, respectively, in *E. coli* is only one of a number of common sequences within the species.

The method presented here proved useful to compare the abundances of mutations occurring within a short genomic region in an entire microbial community. Next generation sequencing techniques provide a cost-effective comprehensive approach to study entire bacterial communities without relying on the cultivation of individual isolates. To distinguish between resistance mutations and inter-species variability, it is important to design the primers as specific for the targeted taxon as possible. If primers are not sufficiently specific, unwanted reads need to be filtered out by matching all reads to the target sequences and discarding reads of low resemblance. However, setting a similarity threshold for such a filtering where you allow for within species variability is not straight-forward. Here, we chose the threshold based on sequence alignments of different *Escherichia* and *Shigella* sequences and closely related genera. Additionally, we propose that amplicon sequencing could also be used to study resistance mutations in the ribosomal rRNA 23S gene that confer resistance to macrolides, lincosamides, and streptogramin B (Vester and Douthwaite, 2001). For future work in finding more suitable markers of fluoroquinolone exposure, we propose a more controlled experimental set up where different environments are dosed with various concentrations of fluoroquinolones for an extended period of time and the effect of the microbiota is studied using metagenomic sequencing.

In this study, we show that there is a significant abundance of fluoroquinolone resistance mutations in environmental *Escherichia* communities residing both in fluoroquinolone-polluted and pristine environments. This suggests that the mutations are not associated with a substantial fitness cost for the bacterium, even where there is no selective pressure for fluoroquinolone resistance. Regardless of whether the detected mutations are sufficient to provide clinically relevant resistance to fluoroquinolones, they are one or two mutations closer to obtaining a high level of resistance.

## Author Contributions

AJ, EK, and JL conceived of the study and participated in its design. AJ collected samples from the Swedish lakes. JF performed the chemical analyses of the sediments. BW prepared DNA from the samples and did the PCR amplifications. AJ designed the PCR primers, performed all data analyses, and drafted the manuscript. All authors read and approved the final manuscript.

## Conflict of Interest Statement

The authors declare that the research was conducted in the absence of any commercial or financial relationships that could be construed as a potential conflict of interest.

## References

[B1] Adachi F. Yamamoto A. Takakura K.-I. Kawahara R. (2013). Occurrence of fluoroquinolones and fluoroquinolone-resistance genes in the aquatic environment. *Sci. Total Environ.* 444 508–514. 10.1016/j.scitotenv.2012.11.077 23291652

[B2] Altschul S. F. Gish W. Miller W. Myers E. W. Lipman D. J. (1990). Basic local alignment search tool. *J. Mol. Biol.* 215 403–410. 10.1016/S0022-2836(05)80360-2 2231712

[B3] Bagel S. Hullen V. Wiedemann B. Heisig P. (1999). Impact of gyrA and parC mutations on quinolone resistance, doubling time, and supercoiling degree of *Escherichia coli*. *Antimicrob. Agents Chemother.* 43 868–875. 10103193 10.1128/aac.43.4.868 PMC89219

[B4] Bhullar K. Waglechner N. Pawlowski A. Koteva K. Banks E. D. Johnston M. D. (2012). Antibiotic resistance is prevalent in an isolated cave microbiome. *PLoS ONE* 7:e34953 10.1371/journal.pone.0034953 PMC3324550 22509370

[B5] D’Costa V. M. Mcgrann K. M. Hughes D. W. Wright G. D. (2006). Sampling the antibiotic resistome. *Science* 311 374–377. 10.1126/science.1120800 16424339

[B6] ECDC (2013). *Antimicrobial Resistance Surveillance in Europe 2012.* Annual Report of the European Antimicrobial Resistance Surveillance Network (EARS-Net). Stockholm: European Centre for Disease Prevention and Control.

[B7] ECDC (2014). *Antimicrobial Resistance Surveillance in Europe 2013.* Annual Report of the European Antimicrobial Resistance Surveillance Network (EARS-Net). Stockholm: European Centre for Disease Prevention and Control.

[B8] Edgar R. C. (2004). MUSCLE: multiple sequence alignment with high accuracy and high throughput. *Nucleic Acids Res.* 32 1792–1797. 10.1093/nar/gkh340 15034147 PMC390337

[B9] EUCAST. (2014). *Breakpoint Tables for Interpretation of MICs and Zone Diameters. Version 4.0: The European Committee on Antimicrobial Susceptibility Testing.* Available at: http://www.eucast.org

[B10] Fick J. Lindberg R. H. Kaj L. Brorström-Lundén E. (2011). *Results from the Swedish National Screening Programme 2010. Subreport 3. Pharmaceuticals.* Stockholm: Swedish Environmental Research Institute Ltd.

[B11] Fick J. Soderstrom H. Lindberg R. H. Phan C. Tysklind M. Larsson D. G. J. (2009). Contamination of surface, ground, and drinking water from pharmaceutical production. *Environ. Toxicol. Chem.* 28 2522–2527. 10.1897/09-073.1 19449981

[B12] Flach C.-F. Boulund F. Kristiansson E. Larsson D. J. (2013). Functional verification of computationally predicted qnr genes. *Ann. Clin. Microbiol. Antimicrob.* 12 34 10.1186/1476-0711-12-34 PMC4222258 24257207

[B13] Flach C. F. Johnning A. Nilsson I. Smalla K. Kristiansson E. Larsson D. G. J. (2015). Isolation of novel IncA/C and IncN fluoroquinolone resistance plasmids from an antibiotic-polluted lake. *J. Antimicrob. Chemother.* 70 2709–2717. 10.1093/jac/dkv167 26124213

[B14] Gaze W. H. Krone S. M. Larsson D. G. J. Li X. Z. Robinson J. A. Simonet P. (2013). Influence of humans on evolution and mobilization of environmental antibiotic resistome. *Emerg. Infect. Dis.* 19 e120871 10.3201/eid1907.120871 PMC3713965 23764294

[B15] Gillespie J. J. Wattam A. R. Cammer S. A. Gabbard J. Shukla M. P. Dalay O. (2011). PATRIC: the comprehensive bacterial bioinformatics resource with a focus on human pathogenic species. *Infect. Immun.* 79 4286–4298. 10.1128/IAI.00207-11 21896772 PMC3257917

[B16] Girardi C. Greve J. Lamshöft M. Fetzer I. Miltner A. Schäffer A. (2011). Biodegradation of ciprofloxacin in water and soil and its effects on the microbial communities. *J. Hazard. Mater.* 198 22–30. 10.1016/j.jhazmat.2011.10.004 22036930

[B17] Gullberg E. Cao S. Berg O. G. Ilbäck C. Sandegren L. Hughes D. (2011). Selection of resistant bacteria at very low antibiotic concentrations. *PLoS Pathog.* 7:e1002158 10.1371/journal.ppat.1002158 PMC3141051 21811410

[B18] Hopkins K. L. Davies R. H. Threlfall E. J. (2005). Mechanisms of quinolone resistance in *Escherichia coli* and *Salmonella*: recent developments. *Int. J. Antimicrob. Agents* 25 358–373. 10.1016/j.ijantimicag.2005.02.006 15848289

[B19] Ishii S. Sadowsky M. J. (2008). *Escherichia coli* in the environment: implications for water quality and human health. *Microbes Environ.* 23 101–108. 10.1264/Jsme2.23.101 21558695

[B20] Kristiansson E. Fick J. Janzon A. Grabic R. Rutgersson C. Weijdegård B. (2011). Pyrosequencing of antibiotic-contaminated river sediments reveals high levels of resistance and gene transfer elements. *PLoS ONE* 6:e17038 10.1371/journal.pone.0017038 PMC3040208 21359229

[B21] Kümmerer K. (2009). Antibiotics in the aquatic environment-A review-Part I. *Chemosphere* 75 417–434. 10.1016/j.chemosphere.2008.11.086 19185900

[B22] Kunz A. N. Begum A. A. Wu H. D’ambrozio J. A. Robinson J. M. Shafer W. M. (2012). Impact of fluoroquinolone resistance mutations on gonococcal fitness and in vivo selection for compensatory mutations. *J. Infect. Dis.* 205 1821–1829. 10.1093/infdis/jis277 22492860 PMC3415892

[B23] Larsson D. G. J. De Pedro C. Paxeus N. (2007). Eﬄuent from drug manufactures contains extremely high levels of pharmaceuticals. *J. Hazard. Mater.* 148 751–755. 10.1016/j.jhazmat.2007.07.008 17706342

[B24] Lindberg R. H. Wennberg P. Johansson M. I. Tysklind M. Andersson B. A. (2005). Screening of human antibiotic substances and determination of weekly mass flows in five sewage treatment plants in Sweden. *Environ. Sci. Technol.* 39 3421–3429. 10.1021/es048143z 15952345

[B25] Lukjancenko O. Wassenaar T. M. Ussery D. W. (2010). Comparison of 61 sequenced *Escherichia coli* genomes. *Microb. Ecol.* 60 708–720. 10.1007/s00248-010-9717-3 20623278 PMC2974192

[B26] Luo N. Pereira S. Sahin O. Lin J. Huang S. Michel L. (2005). Enhanced in vivo fitness of fluoroquinolone-resistant *Campylobacter jejuni* in the absence of antibiotic selection pressure. *Proc. Natl. Acad. Sci. U.S.A.* 102 541–546. 10.1073/pnas.0408966102 15634738 PMC545549

[B27] Machuca J. Briales A. Labrador G. Díaz-De-Alba P. López-Rojas R. Docobo-Pérez F. (2014). Interplay between plasmid-mediated and chromosomal-mediated fluoroquinolone resistance and bacterial fitness in *Escherichia coli*. *J. Antimicrob. Chemother.* 69 3203–3215. 10.1093/jac/dku308 25139837

[B28] Mandal J. Acharya N. S. Buddhapriya D. Parija S. C. (2012). Antibiotic resistance pattern among common bacterial uropathogens with a special reference to ciprofloxacin resistant *Escherichia coli*. *Indian J. Med. Res.* 136 842–849. 23287133 PMC3573607

[B29] Marcusson L. L. Frimodt-Møller N. Hughes D. (2009). Interplay in the selection of fluoroquinolone resistance and bacterial fitness. *PLoS Pathog.* 5:e1000541 10.1371/journal.ppat.1000541 PMC2714960 19662169

[B30] Margulies M. Egholm M. Altman W. E. Attiya S. Bader J. S. Bemben L. A. (2005). Genome sequencing in microfabricated high-density picolitre reactors. *Nature* 437 376–380. 10.1038/nature03959 16056220 PMC1464427

[B31] Morgan-Linnell S. K. Boyd L. B. Steffen D. Zechiedrich L. (2009). Mechanisms accounting for fluoroquinolone resistance in *Escherichia coli* clinical isolates. *Antimicrob. Agents Chemother.* 53 235–241. 10.1128/AAC.00665-08 18838592 PMC2612180

[B32] Peterson J. D. Umayam L. A. Dickinson T. Hickey E. K. White O. (2001). The comprehensive microbial resource. *Nucleic Acids Res.* 29 123–125. 10.1093/nar/29.1.123 11125067 PMC29848

[B33] Pruden A. Larsson D. G. J. Amézquita A. Collignon P. Brandt K. K. Graham D. W. (2013). *Management Options for Reducing the Release of Antibiotics and Antibiotic Resistance Genes to the Environment. Environmental Health Perspectives.* Research Triangle Park, NC: National Institute of Environmental Health Sciences (NIEHS). 10.1289/ehp.1206446 PMC3734499 23735422

[B34] Rice P. Longden I. Bleasby A. (2000). EMBOSS: the European molecular biology open software suite. *Trends Genet.* 16 276–277. 10.1016/S0168-9525(00)02024-2 10827456

[B35] Robicsek A. Jacoby G. A. Hooper D. C. (2006). The worldwide emergence of plasmid-mediated quinolone resistance. *Lancet Infect. Dis.* 6 629–640. 10.1016/S1473-3099(06)70599-0 17008172

[B36] Rozen S. Skaletsky H. (2000). Primer3 on the WWW for general users and for biologist programmers. *Methods Mol. Biol.* 132 365–386. 10547847 10.1385/1-59259-192-2:365

[B37] Ruiz J. (2003). Mechanisms of resistance to quinolones: target alterations, decreased accumulation and DNA gyrase protection. *J. Antimicrob. Chemother.* 51 1109–1117. 10.1093/jac/dkg222 12697644

[B38] Rutgersson C. Fick J. Marathe N. Kristiansson E. Janzon A. Angelin M. (2014). Fluoroquinolones and qnr genes in sediment, water, soil, and human fecal flora in an environment polluted by manufacturing discharges. *Environ. Sci. Technol.* 48 7825–7832. 10.1021/es501452a 24988042

[B39] Untergasser A. Nijveen H. Rao X. Bisseling T. Geurts R. Leunissen J. A. M. (2007). Primer3Plus, an enhanced web interface to Primer3. *Nucleic Acids Res.* 35 W71–W74. 10.1093/nar/gkm306 17485472 PMC1933133

[B40] Vester B. Douthwaite S. (2001). Macrolide resistance conferred by base substitutions in 23S rRNA. *Antimicrob. Agents Chemother.* 45 1–12. 10.1128/AAC.45.1.1-12.2001 11120937 PMC90232

[B41] Waters B. Davies J. (1997). Amino acid variation in the GyrA subunit of bacteria potentially associated with natural resistance to fluoroquinolone antibiotics. *Antimicrob. Agents Chemother.* 41 2766–2769. 9420056 10.1128/aac.41.12.2766 PMC164206

[B42] Wellington E. M. Boxall A. Cross P. Feil E. J. Gaze W. H. Hawkey P. M. (2013). The role of the natural environment in the emergence of antibiotic resistance in Gram-negative bacteria. *Lancet Infect. Dis.* 13 155–165. 10.1016/S1473-3099(12)70317-1 23347633

[B43] Yoshida H. Bogaki M. Nakamura M. Yamanaka L. M. Nakamura S. (1991). Quinolone resistance-determining region in the DNA gyrase gyrB gene of *Escherichia coli*. *Antimicrob. Agents Chemother.* 35 1647–1650. 10.1128/AAC.35.8.1647 1656869 PMC245234

[B44] Zurfluh K. Abgottspon H. Hächler H. Nüesch-Inderbinen M. Stephan R. (2014). Quinolone resistance mechanisms among extended-spectrum beta-lactamase (ESBL) producing *Escherichia coli* isolated from rivers and lakes in Switzerland. *PLoS ONE* 9:e95864 10.1371/journal.pone.0095864 PMC3995870 24755830

