# Supplementary material for: Resistance Mutations in gyrA and parC are Common in Escherichia Communities of both Fluoroquinolone-Polluted and Uncontaminated Aquatic Environments
Source: Front Microbiol. 2015 Dec 9;6:1355. doi: 10.3389/fmicb.2015.01355 (PMC4673309; doi:10.3389/fmicb.2015.01355)
Supplement: Supplementary file 1 [file Table_1.DOCX]

***Supplemental material***

**Figure S1.** a-j) Relative abundance of amino acid substitution in GyrA in each sample. The samples have been arranged in respect to total measured fluoroquinolone concentration in decreasing order.

**Figure S2.** a-j) Relative abundance of amino acid substitution in ParC in each sample. The samples have been arranged in respect to total measured fluoroquinolone concentration in decreasing order.

| **TABLE S1.** Chemical analysis of the sediment samples | **Ref.** | Kristiansson et al. 2011 | | | | | | | This study | | | * All concentrations are given in µg/g organic matter. Detection limit: 0.02 µg/g organic matter. Standard deviation is given within parenthesis. N.D., not detected.  CIP, ciprofloxacin; ENR, enrofloxacin; PEF, pefloxacin; OFL, ofloxacin; LOM, lomefloxacin; DIF, difloxacin; ENO, enoxacin; NOR, norfloxacin. |
| --- | --- | --- | --- | --- | --- | --- | --- | --- | --- | --- | --- | --- |
|  | **Total** | 1 031 | 300 | 71.7 | 25.9 | 16.6 | N.D. | N.D. | N.D. | N.D. | N.D. |  |
|  | **NOR*** | N.D. | N.D. | N.D. | N.D. | N.D. | N.D. | N.D. | N.D. | N.D. | N.D. |  |
|  | **ENO*** | N.D. | N.D. | N.D. | N.D. | N.D. | N.D. | N.D. | N.D. | N.D. | N.D. |  |
|  | **DIF*** | N.D. | N.D. | N.D. | N.D. | 1.52 (1.0) | N.D. | N.D. | N.D. | N.D. | N.D. |  |
|  | **LOM*** | N.D. | N.D. | N.D. | 0.844 (0.33) | 3.75 (3.6) | N.D. | N.D. | N.D. | N.D. | N.D. |  |
|  | **OFL*** | 1.22 (0.67) | 1.86 (2.0) | 3.55 (3.3) | N.D. | N.D. | N.D. | N.D. | N.D. | N.D. | N.D. |  |
|  | **PEF*** | 12.4 (21) | 11.0 (4.7) | 4.72 (1.9) | 3.98 (3.7) | 6.00 (1.4) | N.D. | N.D. | N.D. | N.D. | N.D. |  |
|  | **ENR*** | 102 (40) | 34.5 (4.3) | 11.5 (3.7) | 4.61 (5.3) | 9.42 (1.4) | N.D. | N.D. | N.D. | N.D. | N.D. |  |
|  | **CIP*** | 914 (91) | 253 (99) | 52.0 (16) | 7.15 (3.1) | 5.24 (4.2) | N.D. | N.D. | N.D. | N.D. | N.D. |  |
|  | **Sample** | 1 | 2 | 3 | 1 | 2 |  |  | 1 | 2 | 3 |  |
|  | **Site** | Downstream | | | Upstream | | Downstream | Upstream | Lake |  |  |  |
|  | **Country** | India | | | | | Sweden | | |  |  |  |

| **TABLE S2a.** Relative abundance of common amino acid substitution in GyrA | | | | | |
| --- | --- | --- | --- | --- | --- |
| **Country** | **Site** | **Sample** | **S83L** | **D87N** | **Double** |
| India | Downstream | 1 | 97% | 62% | 61% |
|  |  | 2 | 84% | 36% | 34% |
|  |  | 3 | 69% | 30% | 29% |
|  | Upstream | 1 | 93% | 1% | 1% |
|  |  | 2 | 71% | 28% | 27% |
| Sweden | Downstream |  | 80% | 33% | 32% |
|  | Upstream |  | 89% | 41% | 40% |
|  | Lake | 1 | 87% | 31% | 31% |
|  |  | 2 | 86% | 33% | 32% |
|  |  | 3 | 88% | 34% | 33% |

| **TABLE S2b.** Relative abundance of common amino acid substitution in ParC | | | | | | |
| --- | --- | --- | --- | --- | --- | --- |
| **Country** | **Site** | **Sample** | **S80I** | **E84G** | **E84V** | **Double** |
| India | Downstream | 1 | 63% | 22% | 36% | 58% |
|  |  | 2 | 49% | 0.1% | 8% | 8% |
|  |  | 3 | 0.4% | 0.4% | 0.2% | 0.4% |
|  | Upstream | 1 | 15% | 3% | 2% | 3% |
|  |  | 2 | 22% | 2% | 2% | 4% |
| Sweden | Downstream |  | 49% | 0.5% | 7% | 7% |
|  | Upstream |  | 61% | 0.2% | 17% | 17% |
|  | Lake | 1 | 44% | 0.2% | 4% | 4% |
|  |  | 2 | 34% | 1% | 3% | 4% |
|  |  | 3 | 44% | 0.2% | 8% | 8% |
